# From Pre-Diabetes to Diabetes: Diagnosis, Treatments and Translational Research

**DOI:** 10.3390/medicina55090546

**Published:** 2019-08-29

**Authors:** Radia Marium Modhumi Khan, Zoey Jia Yu Chua, Jia Chi Tan, Yingying Yang, Zehuan Liao, Yan Zhao

**Affiliations:** 1 School of Chemical and Biomedical Engineering, Nanyang Technological University, 62 Nanyang Drive, Singapore 637459, Singapore; 2 School of Physical and Mathematical Sciences, Nanyang Technological University, 21 Nanyang Link, Singapore 637371, Singapore; 3 School of Biological Sciences, Nanyang Technological University, 60 Nanyang Drive, Singapore 637551, Singapore; 4 Tongji University School of Medicine, Shanghai 201204, China; 5 Department of Medical Epidemiology and Biostatistics, Karolinska Institutet, 171 65 Solna, Sweden; 6 Department of Microbiology, Tumor, and Cell Biology (MTC), Karolinska Institutet, Biomedicum, Solnavägen 9, SE-17177 Stockholm, Sweden

**Keywords:** diabetes, pre-diabetes, insulin resistance, hyperglycemia

## Abstract

Diabetes, a silent killer, is one of the most widely prevalent conditions of the present time. According to the 2017 International Diabetes Federation (IDF) statistics, the global prevalence of diabetes among the age group of 20–79 years is 8.8%. In addition, 1 in every 2 persons is unaware of the condition. This unawareness and ignorance lead to further complications. Pre-diabetes is the preceding condition of diabetes, and in most of the cases, this ultimately leads to the development of diabetes. Diabetes can be classified into three types, namely type 1 diabetes, type 2 diabetes mellitus (T2DM) and gestational diabetes. The diagnosis of both pre-diabetes and diabetes is based on glucose criteria; the common modalities used are fasting plasma glucose (FPG) test and oral glucose tolerance test (OGTT). A glucometer is commonly used by diabetic patients to measure blood glucose levels with fast and rather accurate measurements. A few of the more advanced and minimally invasive modalities include the glucose-sensing patch, SwEatch, eyeglass biosensor, breath analysis, etc. Despite a considerable amount of data being collected and analyzed regarding diabetes, the actual molecular mechanism of developing type 2 diabetes mellitus (T2DM) is still unknown. Both genetic and epigenetic factors are associated with T2DM. The complications of diabetes can predominantly be classified into two categories: microvascular and macrovascular. Retinopathy, nephropathy, and neuropathy are grouped under microvascular complications, whereas stroke, cardiovascular disease, and peripheral artery disease (PAD) belong to macrovascular complications. Unfortunately, until now, no complete cure for diabetes has been found. However, the treatment of pre-diabetes has shown significant success in preventing the further progression of diabetes. To prevent pre-diabetes from developing into T2DM, lifestyle intervention has been found to be very promising. Various aspects of diabetes, including the aforementioned topics, have been reviewed in this paper.

## 1. Introduction

Diabetes mellitus (DM) is a form of metabolic disorder whereby the patients suffer high blood sugar levels because their bodies do not respond to, or produce inadequate, insulin—a hormone that helps to stabilize the blood sugar (glucose) level by directing the cells to take up glucose and inhibit hepatic glucose production [1]. There are generally 3 types of diabetes: Type I, II and gestational diabetes but more emphasis is placed on the first 2 types of diabetes. Type I diabetes is a form of autoimmune disease whereby the cells that produce insulin are destroyed by its own immune system whereas type II diabetes, being more common than type I, happens when the body does not respond to the insulin produced. In chronic conditions, diabetes can lead to long-term damage, dysfunction, and failure of different organs, especially the eyes, kidneys, nerves, heart, and blood vessels [2,3]. A few typical complications of diabetes are blindness (retinopathy), renal failure, diabetic foot disorders (severe infections on the legs, which will eventually lead to amputation) and cardiovascular diseases [4].

Despite the tremendous effort put into prolonging the lives of patients with DM, diabetes has remained as the 5th leading cause of death worldwide and has directly resulted in 1.6 million deaths [5,6]. Diabetic patients are reported to have a 15% increased risk of premature death and life expectancy reduced by approximately 10 and 20 years for type I and type II diabetes, respectively [7,8]. According to the 2017 International Diabetes Federation (IDF) statistics, the global prevalence of diabetes among the age group of 20–79 years is 8.8%. In addition, 1 in every 2 persons is unaware of the condition [9]. By 2030, diabetes is estimated to affect 439 million adults, up from the previous estimation of 366 million [10,11]. However, statistics show that the number of diabetic patients worldwide was 422 million in 2014 [6]. Thus, a new report has estimated that there will be at least 592 million diabetes cases worldwide in 2035 [12]. Diabetes is also reported to be more prevalent in the urban population when compared to the rural population, and affects more men than women [13]. Diabetes can affect anyone, but many studies that date back to as early as 1969 show that Asians, people who origin from Far East, Southeast Asia, or the Indian subcontinent, particularly South Asian, are more susceptible to diabetes than people from other ethnicities [14,15,16,17,18,19].

A study conducted in 1985 at Southall, West London, demonstrated that the prevalence of diabetes in Asians was at least 3.8-fold higher than Europeans and 5-fold higher for patients aged between 40 to 64 years old [20]. Ramachandran et al., published in 2012, stated that more than 60% of the world’s diabetic population were from Asian countries, as Asians are genetically and ethnically predisposed to diabetes and thus have a lower tolerance for environmental risk factors. The paper reported that Asians develop diabetes at a lower body mass index (BMI) as well as a smaller waist circumference, as compared to the Western population, and have a younger onset age for diabetes [21]. This is mirrored in a study conducted in the United States, where Asian Americans were shown to be at a higher risk of getting diabetes, despite having lower rates of obesity than non-Hispanic whites [22]. Another study of a total combined sample size of 54,326 people was also conducted in the United States. In this large-scale study, the prevalence of diabetes in foreign-born South Asians (13.6%) was almost 2 times more than foreign-born other Asians (7.4%), and more than twice that of U.S.-born non-Hispanic whites (5.6%). Among the patients with normal BMI, the prevalence in foreign-born South Asians was at least 5-fold higher than U.S.-born non-Hispanic whites at 14.1% and 2.7%, respectively. Although foreign-born South Asians have a prevalence similar to that of U.S.-born Hispanics, only 45.6% of the foreign-born South Asian diabetic populations are considered as overweight or obese, compared to 67.1% of the U.S.-born Hispanics [23]. This is in line with the claim made by Ramachandran et al.

As diabetes is a worldwide epidemic, and more prevalent in South Asian ethnicity, Asian countries will not be spared by this disease, and some would label Asia as the epicenter of this epidemic [24]. The International Diabetes Federation (IDF) reported that in 2013, nearly 382 million people worldwide suffered from diabetes and 60% of them reside in Asia [25]. IDF also predicted that by 2035, the number will go up to 592 million people. In contrast to the statistics found in the Western countries, where diabetes is more common in elderly, research has shown that the Asian population has a high incidence rate of diabetes even in young or middle-aged adults [26]. This further supports the idea that people of South Asian ethnicity are more vulnerable towards diabetes. At the same time, there is growing evidence showing that Asian countries are experiencing a faster rate and higher burden of diabetes patients than any other regions [10,27,28,29]. According to a nation-wide survey conducted in Singapore, a total of 440,000 Singaporeans has been diagnosed with diabetes and this number is expected to grow up to 1 million by 2050, which is 15% of the population [30]. This is strong evidence that diabetes is an urgent health concern in Asia, which must be addressed immediately. It is important to raise the awareness of the public and draw attention to the adverse effects of diabetes, as well as gather national support to tackle diabetes.

To be diagnosed as diabetic, one’s blood glucose level needs to be equal to or above a certain value. According to the American Diabetes Association (ADA), there are four methods for the diagnosis of diabetes and the same methods are used for the screening of pre-diabetes in patients [31]. The methods are:

(1) Fasting plasma glucose test (FPG): where fasting refers to the absence of food and drink intake, apart from water, for at least 8 h before the test; or
(2) Oral glucose tolerance test (OGTT): where a patient consumes a glucose syrup solution containing 75 g of glucose before which a blood test is carried out to determine 2-hr plasma glucose (PG); or
(3) A1C (Glycated hemoglobin or hemoglobin bounded to glucose) levels via a laboratory test; or
(4) Random PG of more than or equal to 200 mg/dL or 11.1 mmol/L in patients that displayed symptoms of hyperglycemia or hyperglycemic crisis.

A comparison between results for normal, diabetes and pre-diabetes diagnoses based on the first three criteria can be summarized as in Table 1 [32]. 

There are pros and cons in these different diagnostic methods. FPG ≥ 140 mg/dL is a highly specific but rather insensitive test for the diagnosis of DM [33]. Almost all subjects with FPG ≥ 140 mg/dL will have PG in OGTT ≥ 200 mg/dL, but a significant portion of subjects (depending on the population) with PG in OGTT ≥ 200 mg/dL will not have an FPG ≥ 140 mg/dL. A1C test measures the glycation of proteins and can potentially indicate chronic hyperglycemia, but this test does not directly prove high blood glucose levels—the clinical definition of DM [34]. Furthermore, measuring PG in OGTT is a more accurate method than measuring FPG and A1C in diagnosing DM [35]. However, PG in OGTT ≥ 200 mg/dL may also indicate impaired glucose tolerance, rather than full-blown diabetes. Therefore, it is recommended that more than one test is used in diagnosing DM.

Many studies, as reported above, have been done on diabetes patients. However, little is known about the pre-diabetic conditions. Pre-diabetes is a term used to describe the buffer period before the onset of type II diabetes, where the blood sugar level is higher than normal but lower than the diagnostic criteria of type II diabetes. Impaired β-cell function and increased insulin resistance are two pathological pathways that lead to pre-diabetes, and subsequently, diabetes. The onset of increased insulin resistance starts years before diabetes and even pre-diabetes [36]. More specifically, insulin resistance in skeletal muscle tissues can be regarded as the initiating factor that is present decades before impaired β-cell function [37]. Additionally, in a study conducted by Cerasi et. al., it was observed that there was a decrease in glucose-induced insulin release of the pancreatic β-cells in diabetic and pre-diabetic patients [38]. The dose–response curve for glucose-induced insulin release shifted to the right and further right for pre-diabetic and diabetic patients, respectively, as compared to normal individuals [38]. Moreover, it was also reported that there was a significant increase in β-cell function 3 to 4 years before diabetes diagnosis, followed by a steep decrease [36]. Combining the continued increase in insulin resistance with a decrease in β-cell function, glucose levels in the blood become unregulated and pre-diabetes then evolves into full-blown diabetes. 

It has been estimated that the number of pre-diabetic cases will increase to more than 470 million people worldwide, and this is understandably a worrying trend [39]. The Centers for Disease Control and Prevention (CDC) reported that in 2015, almost half (48.3%) of the adult population aged 65 and above had pre-diabetic conditions and that approximately 84.1 million people in the U.S. were already pre-diabetic. In accordance with the higher prevalence of diabetes in South Asian ethnicity, the Asian population indeed showed a higher prevalence in pre-diabetes than the Western population [40]. Studies have shown that non-East Asian countries such as Saudi Arabia (6.8%), India (6.3%) and South Latin America (17.8%) have pre-diabetes prevalence 2 to 5-fold lower than East Asian countries as China (35.7%). A total of 37% of pre-diabetic patients who leave their condition untreated may see themselves develop diabetes in 4 years [13,41,42,43,44,45]. Moreover, if lifestyle changes were adopted, long-term studies have shown that the risk of this progression—from pre-diabetes to diabetes—can be lowered for an extended period of 10 years [41]. Pre-diabetes is seen as the critical phase, because studies have shown that at this stage, the condition is reversible and could serve as a potential route to combat diabetes [41,45]. Thus, in this paper, we conduct a general review which includes pre-diabetic conditions, by pooling together publications that discuss diabetes from different perspectives, in the hope that, with a better understanding of this disease, we can provide invaluable insight as to how we can combat diabetes around the world.

## 2. Risk Factors of Type II Diabetes

Diabetes is one of the most prevalent diseases worldwide. Even though a considerable amount of data has been collected and analyzed regarding diabetes, the actual molecular mechanism of developing type 2 diabetes mellitus (T2DM) is still unknown. Although the mechanism behind this disease has yet to be fully understood, according to most researchers, certain factors play a key role in driving the onset of T2DM. Both genetic and epigenetic factors are associated with T2DM.

### 2.1. Evidence for Lifestyle and Environmental Risk Factors

#### 2.1.1. Obesity

T2DM occurs due to various factors that cause insulin resistance and β-cell dysfunction. Various cross-sectional and prospective studies have unanimously confirmed that obesity is one of the environmental factors that has a correlation with T2DM. A total of 50% of people with T2DM are obese (BMI > 30 kg/m^2^). A total of 90% of diabetic patients are overweight (BMI > 25 kg/m^2^). Therefore, even just a moderate weight loss can have an immense impact in controlling diabetes [46,47]. Due to obesity, the levels of adipocytes, cytokines (interleukin-1 (IL-1) and interleukin-6 (IL-6)), and tumor necrosis factor alpha (TNFα)) increases in the body. The increased amount of these components triggers a signaling pathway which represents an inflammatory action of the adipose tissue [48]. This chronic low-grade inflammatory action might promote insulin resistance in cells [49]. Indeed, in a study conducted by Barbarroja et al., it was found that the level of mRNA expression of IL-1β and IL-6 among insulin resistant obese people was high in comparison to non-insulin resistant obese patients [48]. In a study conducted on the health of nurses, as reported by Wild and Byrne, it was found that women whose BMI was greater than 35 had a 49-times more chance of developing T2DM compared to those whose BMI was less than 22. Studies on men have shown similar results. Wild and Byrne also reported that men with a BMI greater than or equal to 35, had a 42-times higher chance of developing the disease than men with a BMI less than 23. This study was conducted on a cohort of men from the United States of America. For Asians (mostly Indians), the onset of higher risk of diabetes starts at an even lower BMI value (15–20). However, recent studies have shown that larger waist circumference could be a better indication of developing T2DM than BMI. Several studies in China, the U.S., and Finland state that the risk of developing T2DM can be lowered through decent weight loss [50]. 

#### 2.1.2. Sedentary Lifestyle

Through various studies, a sedentary lifestyle has been proven to be one of the major causes of developing T2DM. Watching television is one of the major sedentary activities. Watching television is worse than any other sedentary activity, such as playing board games, sewing, reading, writing or driving a car. This is because the metabolic rate while watching television is the lowest among the mentioned sedentary activities [51]. A meta-analysis was conducted with 10 studies, which consisted of 505,045 participants in total. In the analysis, it was found that people with longer TV time had a 112% higher pool for T2DM compared to the people with shorter TV time [52]. The explanation for this can be found in BMI. As people tend to spend more time watching TV, they are less physically active. Hence, their BMI increases. The physiological reasoning is that during acute contraction of active muscles, there is an immediate uptake of plasma glucose, and when someone is physically inactive, this does not happen often enough. The decrease in insulin sensitivity is another reason. A study was conducted by Balkau et al. with 801 apparently healthy volunteers, to find a relation between sedentary time and insulin sensitivity. In the study, sedentary time was measured with accelerometry and insulin sensitivity was measured with a hyperinsulinemic-euglycemic clamp. The study concluded that there was an inverse relationship between sedentary time and insulin sensitivity. The difference between the most and least physically active hours was only 4 h; however, the insulin sensitivity ranged around 40% [53]. Therefore, by replacing just 30 min of sedentary lifestyle with moderate to vigorous physical activities, it can improve insulin sensitivity by 15% [54]. Moreover, since a sedentary lifestyle is strongly correlated with weight gain or obesity, the pathophysiological paths of obesity causing diabetes may also be applicable to a sedentary lifestyle.

#### 2.1.3. Ageing

A considerable number of studies have found that the chances of T2DM increase with the increase in age. According to the National Diabetes Statistics Report of the United States of America, it was found that about 4.0% of people had diabetes in the age group 18–44 years. This number increased to 17.0% in the age group 45–64 years, and the percentage further rose to 25.2% for the age group ≥65 years [55]. Similar results were found in a survey conducted in England. According to the Health Survey for England (2006), the highest prevalence of diabetes existed in the age group 65–74 years. A total of 15.7% of the surveyed men in that age group had diabetes, whereas, in the same age group, 10.4% of women had diabetes [56]. In another study conducted by Suastika et al. on a cohort in Bali, it was found that the prevalence of T2DM was more than 2-fold in the older generation compared to younger people [57]. A study was conducted on elderly Chinese in 2000 in Taiwan. In the study, Peng et al. found that 16.9% of them had T2DM. After 5 years, a follow-up survey was done on the same group of people and it was found that the prevalence of diabetes rose to 23.7% [58]. The most likely pathophysiological reason behind this is that the human body gets less sensitive to insulin as it ages. In addition to this, the β-cells get altered or show insufficiency in insulin production as the human body ages [59]. 

#### 2.1.4. Sex and Gender

According to a report published by International Diabetes Federation (IDF) in 2017, the total number of adults (20–79 years) diagnosed for T2DM was 425 million, which is 8.3% of the total population. The distribution was not equal between men and women. There were 17.1 million more men diagnosed with diabetes than women (221.0 million were men and the rest, 203.9 million, were women). This number, both men and women together, is expected to rise to as high as 629 million by the year 2045, which is 48% more than in 2017 [9]. The imbalance in the prevalence of diabetes based on gender is yet to be understood. Though more men are diagnosed with T2DM, women have more cases of obesity which is one of the major causes of T2DM. The probability of diabetes according to sex and gender arises from various biological and environmental factors. In endocrinology, the most prominent effect of sexual dimorphisms is expressed through T2DM. This might be due to the difference in sex chromosomes, sex-specific gene expression of autosomes, sex hormones and their effects on organ systems. Anatomically, men and women have different body fat distribution and brown adipose tissue (BAT). The healthy range of BMI value of men and women is also different. The onset of risk of diabetes starts at a lower BMI value for men compared to women. [60]. Conversely, women have more obesity induced diabetes compared to men. In terms of fat distribution, men have more trunk and visceral fat (VAT), and liver fat, in comparison to women of the same BMI and age [61,62]. Moreover, men have a higher amount of VAT for any amount of total fat [63]. Women have more deposition in leg fat tissue [64]. This difference in fat distribution might be one of the underlying reasons for the difference in diabetes prevalence among the genders. Recent studies have found that the different level and activity of brown adipose tissue (BAT) in the genders may play a role as well [65]. There is a negative relation between the activity of BAT and diabetes risk. Increased activity of BAT reverses obesity, increases adiponectin and reduces insulin resistance [66]. Besides all of the aforementioned reasons, the difference in diabetes pattern among the genders may also be due to different exposures to environmental factors, such as nutrition, health care facilities for prevention or treatment of diseases, lifestyle, socioeconomic status, psychosocial stress, sleep deprivation, work stress and many more [67,68,69]. The extent of the effect of the factors is different for males and females. 

#### 2.1.5. Hypertension

The cases of diabetes and hypertension overlap significantly. Therefore, it is very difficult to understand if diabetes causes hypertension, or if hypertension causes diabetes. Recently, the American Heart Association regarded diabetes to be a risk, rather than a risk factor, of coronary heart diseases after recent studies showed that the risk of having myocardial infarction in diabetic individuals is equal to that of patients who had a history of previous myocardial infarction [70]. Nonetheless, Cheung and Li have suggested that both the diseases might carry the same or similar etiology and disease mechanisms [71]. In the Hong Kong Cardiovascular Risk Factor Prevalence Study, Cheung found that only 42% of the diabetes patients had normal blood pressure and 56% of the hypertension patients had normal blood glucose level [72]. Cheung and Li have concluded that both diabetes and hypertension are the results of a metabolic syndrome which is caused by obesity [71]. Therefore, obesity is a risk factor for both diseases. 

#### 2.1.6. Smoking

Several long-term studies have concluded that people who are chronic smokers have a higher risk of developing T2DM as compared to nonsmokers [73,74,75,76]. In one of the studies, it was found that people having 20 cigarettes a day had 61% higher risk of developing T2DM, whereas, people having less than 20 cigarettes a day had only 29% higher risk of developing T2DM [74]. The difference is evident and has been backed by various studies; a higher dose of insulin was needed to achieve the same metabolic control in smokers as compared to nonsmokers. This risk arises due to the insulin insensitivity resulting from nicotine, one of the active chemicals in cigarettes [77]. Additionally, it is not only smoking, but also nicotine patches, that have been found to decrease the effect of insulin [78]. This further confirms that nicotine is an active compound that causes diabetes. Smoking is found to have severely aggravated glucose tolerance and the insulin sensitivity index [79]. Bergman et al. found that smokers have reduced expression of peroxisome proliferator-activated receptor-gamma (PPAR-γ), a transcription factor that promotes insulin sensitivity. They also found that smokers have increased Serine 636 phosphorylation of insulin receptor substrate IRS-1 [80]. The phosphorylation of this serine residue results in decreased insulin signaling. Among people with normal BMI, studies have found that smokers have higher abdominal obesity than nonsmokers, which is a key risk factor for diabetes [74,81]. Yun et al. conducted a study in which they found that people smoking more than 20 cigarettes had a 1.93 abdominal obesity ratio compared to people who had never smoked [82]. Smoking is linked to causing detrimental changes in body composition which might lead to the development of diabetes [83]. Through research, Yoshikawa et al. have found that there are nicotine receptors on the pancreatic insulin-producing β-cells. Evidence from the same research indicates that, not only does chronic exposure to nicotine increase the risk of T2DM, but also that acute exposure causes a reduction in insulin sensitivity [84]. Results from a study conducted by Bruin et al. have also shown that nicotine could cause pancreatic β-cell apoptosis and a reduction in pancreatic β-cell mass [85]. 

Studies conducted specifically on Asians have also found the positive correlation between smoking and diabetes. For example, such a trend has been reported by studies in China, Taiwan and Korea. The majority of Asian men (50% to 60%) are smokers [86,87,88]. China is both the largest producer and largest consumer of cigarettes in the world, followed by India, another Asian country. More than 33% of cigarettes is consumed by people in China alone [89,90]. In a study conducted on 513 Japanese men, there was a positive correlation between smoking and higher waist-to-hip ratio (visceral adipose: subcutaneous adipose) [83]. Therefore, the impact of smoking on diabetes is likely to be intense among the Asian nations.

#### 2.1.7. Alcohol

Alcohol is another risk factor for T2DM when consumed above a certain threshold value. However, when alcohol is consumed below the threshold value, it reduces the risk of T2DM. The latest meta-analysis to find the relation between alcohol consumption and T2DM was carried out by Knott et al. The meta-analysis reviewed 38 studies consisting of 1,082,639 male subjects and 819,966 female subjects. Knott et al. found that when alcohol was consumed at any amount below 63 g/day, there was a reduction in the risk of T2DM. Moreover, when alcohol consumption was 10–14 g/day, the reduction was highest. To conclude, when alcohol consumption increases above 63 g/day there is a positive correlation with the risk of T2DM [91].

### 2.2. Evidence for Genetic Risk Factors

Genetics is another major risk factor for T2DM. Various studies found that people of certain ethnic groups have higher chances of developing T2DM than people of other ethnic groups. For example, Pima Indians living in Western countries have twice the risk of T2DM than native Europeans. It was found that people whose parents have T2DM have 6 times more risk of developing T2DM, as compared to the control group. Therefore, people with parents who have T2DM, have a 40% higher chance of developing the same condition [92]. Over the past 35 years, numerous studies have been conducted to find if genetics is a reasonable risk factor of T2DM. Park found that family-based linkage analysis, candidate gene approach and genome wide-association studies (GWAS) were the three approaches used to find if genetics is responsible. In total, more than 40 genes have been identified as being responsible for T2DM [93]. 

#### 2.2.1. Family Based Linkage Analysis

As reviewed by Park, in this kind of analysis, chromosomes of members of the same blood relation are studied to find the genetic disposition for T2DM [93]. Of the many studies that have been conducted, a few of them identified *CAPN10* and *ACRP30* as the putative diabetes susceptibility genes [94,95]. However, the results did not show consistency over the entire study population. Hence, these genes could not be definitively concluded as being high risk factors for T2DM [93].

#### 2.2.2. The Candidate Gene Approach

Specific candidate genes or genetic regions of known biological functions are studied. As reported by Altshuler et al. and Gloyn et al. certain genes such as *PPARG* and *KCNJ11* are linked to T2DM [96,97]. The *PPARG* gene is responsible for coding PPAR-γ. This receptor demonstrates insulin sensitivity by controlling the transcription of a few other genes [98]. As suggested by Altshuler et al., when the 12th amino acid (P12A) is replaced with proline, the risk of T2DM increases significantly [96]. PPAR-γ improves insulin sensitivity through regulation of the glucose and lipid metabolism related genes such as glucose tranporter-2 (GLUT-2) and liver type glucokinase (LGK). As GLUT-2 and LGK are responsible for sensing the fluctuation of glucose level in the blood, activation of GLUT-2 and LGK by PPAR-γ may restore the glucose-sensing ability of β-cells [99]. Furthermore, activation of PPAR-γ also upregulates the expression of proteins involved in insulin response (insulin receptor substrate 1 and 2, phosphatidylinositol 3-kinase) and glucose transport (GLUT-4 and c-Cbl-associated protein) [100,101]. Hence, PPAR-γ plays a central role in insulin sensitivity through regulating the genes involved [102]. Apart from PPAR-γ, a polymorphic variation of *KCNJ11* may also significantly increase the risk of T2DM [103]. The pore forming subunit of the ATP sensitive potassium channel (Kir6.2) of the β-cells of the pancreas is coded by this gene. A gain-of-function mutation of this channel makes the β-cells more permeable to potassium, thereby making it difficult for the cells to be depolarized [104]. Florez et al. and Nielsen et al. suggested that when the 23rd amino acid (E23K) of this gene is substituted by glutamic acid, the risk of T2DM increases substantially [105,106]. Therefore, *PPARG* and *KCNJ11* can be claimed to be responsible for T2DM to some extent. Hence, some anti-diabetes medications are developed targeting these genes.

#### 2.2.3. Genome Wide Association Studies (GWAS)

GWAS is the latest of the three approaches in identifying the genetic link of T2DM. Not only have new genomic regions (*SLC30A8*, IGF2BP2, *CDKN2A/2B*, *CDKAL1*, etc.) concerning T2DM been detected through this approach, but the previously found genomic regions (*PPARG* and *KCNJ11*) have also been confirmed. A few of the genes that are claimed to be associated with T2DM have been listed below (Table 2). 

## 3. Novel Methods to Monitor Blood Glucose Level

According to the ADA and the National Institute of Diabetes and Digestive and Kidney Diseases (NIH) of the United States, diabetic patients should carry out checks on their blood glucose levels regularly to ensure that their condition is under control, as both low and high glucose levels can have deleterious effects on the body [113,114]. Although the exact number of times to check depends on the type of diabetes and the patient’s medications, there are four common times suggested by the NIH for carrying out the testing: after waking up (which models fasting), before eating, two hours after eating and before going to sleep [115,116]. Glucose levels should be targeted at 80–130 mg/dL before eating and at less than 180 mg/dL after eating [116]. This testing can be done by the patients themselves, using a blood glucose meter (also known as a glucometer). Briefly, as presented by most glucose meter brochures:

1. Hands should be washed before testing as dirt and residue can lead to inaccuracies.
2. Insert a single test strip into the glucose meter.
3. A small drop of blood should be obtained by prinking the finger-tip using the lancet (needle on blood sampling device), where the volume of blood should be sufficient in filling the test field.
4. Place blood drop onto test strip without smearing it, as smearing can lead to inaccuracies.
5. Glucose meter will then display the blood glucose level.

The glucometer provides diabetic patients with fast and rather accurate measurements of blood glucose levels though there are factors that can lead to inaccuracy such as errors during the application, extreme environmental conditions, and interferences from medication which may thereafter lead to treatment errors [117]. Moreover, such errors can be eliminated through proper storage of blood glucose testing tools and proper education of patients as suggested by Erbach et al. [117] However, what cannot be avoided during the patient’s self-monitoring of blood glucose (SMBC) is the need for finger pricking. Finger pricking, when done several times a day for many years, can not only be painful for patients—making SMBC difficult for children—but also result in the development of massive scarring and callous formation, as well as the loss of sensibility, as previously reviewed by Heinemann [118]. 

In view of this, there have been growing demands for, and numerous developments in, sensors used to measure blood glucose, either directly or indirectly, that do not require finger pricking or that are minimally invasive. These modern methods of glucose sensing have been previously reviewed by Bruen et al. [119] Some of these new methods that were reviewed include a glucose-sensing patch that detects glucose present in the interstitial fluids [120]; SwEatch, a watch platform used for the analysis of sodium present in sweat, in place of a glucose sensor [121]; an eyeglass biosensor that contains both lactate and potassium sensors [122]; breath analysis to detect volatile organic compounds, relating to the detection of acetone [123] as an alternative biomarker for glucose monitoring in diabetes; a lactate sensing mouth guard for the sensing of lactate present in saliva in place of a glucose sensor [124]; and finally, a smart-contact lens developed by Google that could be utilized for the detection of glucose levels present in the ocular fluids [125]. Despite these innovations, however, many are still under development and are stricken with problems, which include sweat contamination [121] and damage to microneedles during penetration for the glucose-sensing patches [119], skin surface contaminations [126] and low production of sweat [127] when sweat analysis is carried out, and lastly, the non-specificity of using acetone as a diabetes biomarker [128] and interferences from other electroactive species present in tears [57].

## 4. Treatment of Pre-Diabetes

Pre-diabetes is a reversible condition. If proper measures are taken during this critical period, then a person can be spared the long-term complications. Pre-diabetes is the early indicator of diabetes that occurs when the patient is diagnosed with impaired glucose tolerance (IGT) and/or impaired fasting glucose (IFG). The onset of both pre-diabetes and diabetes begin with the insulin resistance of cells [129,130,131]. In most cases, at the dawn of T2DM, the individual starts to show insulin insensitivity. To combat this inefficiency of insulin function and maintain normal blood glucose levels, the β-cells of the pancreas start to produce more insulin, which is known as hyperinsulinemia [132]. Two of the major attributes of IGT and IFG are insulin resistance and a decrease in pancreatic β-cell function [132]. Therefore, for the successful treatment of pre-diabetes, these two features must be addressed and resolved promptly before it aggravates to the point when the condition is no longer reversible. 

### 4.1. Lifestyle Intervention

Various studies have suggested that there is a genetic disposition for insulin insensitivity. Certain epigenetic risk factors, such as obesity, lack of physical exercise, and a physically inactive lifestyle worsens the insulin resistance [133,134,135,136]. Treatment of pre-diabetes through lifestyle intervention targets the risk factors such as obesity and diet. Lifestyle intervention mainly comprises regular and nutritious dietary advice, instructions for physical activities and weight loss [137]. Physical activity improves insulin sensitivity by increasing free fatty acid oxidation and improving skeletal muscle mitochondrial function, as well as reducing lipotoxicity in skeletal muscles and the liver [138]. Physical exercise also increases the serum level of adiponectin, which helps to improve insulin sensitivity [139]. Physical activity acts as a physiological stressor which increases glucose uptake by the muscle cells. When the level of physical activity is low, GLUT-4 remains inactive and does not let glucose enter the cell. Conversely, when there is insulin or physical activity, GLUT-4 allows glucose to enter the cell [140]. Other studies have also found that the β-cell function and glucose regulation improve as a result of moderate to intense exercise. This improvement in function is independent of weight loss or obesity [141,142]. However, these studies were not directly done on pre-diabetics, but rather on people intending to prevent diabetes. Nonetheless, the references used in this review paper are justified, because pre-diabetes is the pre-condition of diabetes and most people undergo pre-diabetes before fully developing diabetes. Physical exercises improve β-cell function and insulin sensitivity among pre-diabetics as well [138,143]. Various studies have found that the risk of diabetes reduces by 18% to 40% due to the consumption of a nutritious diet that contains foods with a low glycemic index, such as cereal fiber or a mixture of whole grain and bran [144]. Reducing the consumption of sugar-containing beverages also significantly reduces the risk of diabetes. For example, people drinking more than one sugar-containing beverage per day have 26% more risk of developing diabetes compared to people who have less than one per month [142,145]. Therefore, improved diet can play a major role in preventing the development of diabetes from pre-diabetes.

The Da Qing IGT and Diabetes Study is one of the earliest studies done in Da Qing city, China, on pre-diabetes in order to establish a relation between lifestyle intervention and pre-diabetes progression [146,147]. In the study, 577 pre-diabetics were studied over a period of 6 years. Each of them was assigned to one of four groups (control, improved diet only, exercise only, improved diet with exercise). After 6 years, data showed that 67.7% of people from the control group developed diabetes, whereas, 46.0% of people from the improved diet with exercise group developed diabetes [147]. Ever since then, many studies and surveys have been conducted on a larger number of subjects in different countries all around the world. The United States Diabetes Prevention Program (DPP) and the Finnish Diabetes Prevention Study (DPS) are the two largest diabetes prevention studies which have found promising results in terms of lifestyle intervention. The DPP study was conducted by Knowler et al. (2002). They found that there was a 58% decrease in new diabetes cases after 3 years of intensive lifestyle interventions (ILI). In this study, 3234 pre-diabetic individuals with a mean age 51 years were either given placebo or Metformin: 2 × 850 mg per day, or changes were made in their lifestyles. Lifestyle modification included losing 7% of body weight, reducing 25% of total calorie intake and doing 150 min of physical exercise per week. In the study, it was found that there was a 16% reduction in developing diabetes per 1 kg decrease in weight. The number of participants who developed T2DM per 100 participants were 4.8, 7.8 and 11.0 for lifestyle intervention, Metformin, and placebo respectively. Moreover, in the same study, participants who lost weight and met the required physical activity had the risk of diabetes reduced by more than 90% [133,148]. Therefore, it is evident that weight loss played a principal role in risk reduction. 

Furthermore, in the Finnish DPS conducted by Toumilehto et al., five recommendations were given to the intervening individuals. The benefits of lifestyle intervention were found to be dependent on the extent of the execution of the goals. The goals included total fat intake less than 30% of total energy intake, fiber intake greater than or equal to 15 g per 1000 kcal, saturated-fat intake less than 10 percent of energy intake, weight reduction greater than 5 percent of total body weight, and exercise of more than 4 h/wk. A total of 522 pre-diabetic individuals participated in the study, each of them being assigned to either the control group or the intervention group. Among the intervention individuals, the risk of developing diabetes was 58% lower compared to the control group. Therefore, both the Finnish DPS and DPP were consistent in results [149]. Various studies have been conducted on the Asian population to observe the effect of lifestyle intervention in the treatment of pre-diabetes or T2DM. In the Indian Diabetes Prevention Program (IDPP) whereby people received advice on physical activity, the risk of developing diabetes reduced by 28.5% compared to the control group [142,150]. Another four different studies found that insulin sensitivity can be improved by losing weight and/or exercising [151,152]. Insulin sensitivity improves 30% by losing only 5% of body weight [151]. In a Japanese study conducted with IFG, it was found that intensive intervention has a more positive effect than less intensive intervention [153]. To prevent pre-diabetes developing into T2DM, lifestyle intervention has been found to be very promising. However, maintaining the final reduced weight and continuing exercise is impractical [154]. For instance, the individuals who had participated in the DPP study were found to regain weight after the termination of the study [155]. It is impractical for an individual to continue maintaining a 5% weight loss in real life. Another impediment in the long-term implementation of lifestyle intervention is the lack of patient motivation. To overcome these barriers, a pilot study was done with an advanced version of lifestyle intervention by Pot et al. The program was developed by Foundation Nutrition Alive and was called “Reverse Diabetes2”. Even though this program focused on treating T2DM, the same idea could be applied for treating pre-diabetics. It was a 6-month program and was conducted with T2DM patients who had completed the “Reverse Diabetes2” within February 2015 and March 2016. The additional developments made to the mainstream lifestyle intervention included rigorous counselling on nutrition and lifestyle, digital coaching and education platform, physician guided medication administration, and cooking lessons. In the study, patients were continuously motivated, and were given required knowledge and skills to adapt to the nutritious diet and improved lifestyle. The program was conducted by an army of trained individuals: a dietician, a personal coach and a nurse in alliance with the patient’s general practitioner. The program mainly focused on giving dietary advice to patients. Unlike the more prevalent and common lifestyle intervention programs, patients were given the liberty to choose their preferred dietary option in this program. The participating members of this study were assigned in groups. In total, 73 individuals in four groups completed the program. Members of each group were well connected through social network platforms such as Facebook and WhatsApp, so that they could motivate each other to continue the intervention. The results of this study revealed that the new approach to lifestyle intervention led to improved glucose control of the T2DM patients. In the study, there was further evidence of a reduction in consumption of glucose lowering medication when proper lifestyle intervention was conducted. Reducing or completely stopping the glucose lowering medication was medically justified in 49% of the participating individuals. Furthermore, in this program, the average weight loss was 4.9 kg in 6 months [156]. This study again confirms that weight reduction plays a role in reversing T2DM. Similar effects can also be expected for reversing pre-diabetes. Therefore, this new approach can be used while implementing the recommendation for lifestyle intervention in treatment of pre-diabetes. This could be an indication that lifestyle intervention, if properly implemented, is a better method for controlling pre-diabetes or diabetes than pharmacological intervention. However, although no major research has been carried out to date, investigating the biological mechanism behind this success, it can be concluded from the large amount of study data that lifestyle intervention can, in fact, treat the progression of pre-diabetes to T2DM, to a significant extent. More in-depth and rigorous research needs to be done in this field to find the actual mechanism of action of this success. Even though a satisfying number of studies have been conducted in different countries to find the link between lifestyle intervention and diabetes and pre-diabetes control, no nationwide lifestyle intervention program has been successfully implemented by the government. Many countries have taken several steps to control diabetes through lifestyle intervention, but very few have addressed pre-diabetes, which is potentially more reversible than T2DM. 

For example, the Singapore government is carrying out various intervention programs as part of “Singapore’s War on Diabetes” to make its people more aware of diabetes and pre-diabetes. The program also aims to train the citizens to fight these conditions. For example, in July 2017, to manage pre-diabetes, the Ministry of Health (MOH) published the Appropriate Care Guide (ACG). This guide contains advice on lifestyle intervention. An initiative by the Health Promotion Board (HPB) is the 12-week Diabetes Prevention Program (DPP), which consists of 2 nutrition workshops, 9 exercise lessons and 1 goal setting workshop. Monthly short message service (SMS) alerts are sent to people regarding health tips. Moreover, HPB has launched the HealthHub Track app which helps its users to track physical activity levels. One of the latest additions to HealthHub is the Diabetes Risk Assessment (DRA) tool, which helps young adults to determine whether they should go for diabetes screening or not. MOH has heavily subsided diabetes screening under the Screen for Life (SFL) program [157]. However, even after all these steps, there is no guarantee that pre-diabetes can be completely treated, as most people are unaware that they have pre-diabetes. Also, no formal nationwide study has been conducted to know whether most Singaporeans are aware of the facilities provided by government and how successfully all of these have been implemented. More interactive approaches need to be undertaken in Singapore and the maximum output of the existing facilities need to be ensured. Nonetheless, lifestyle intervention is one of the most reasonable and safe ways to prevent the progression of pre-diabetes to T2DM [137]. Other risk factors of diabetes such as smoking can also be included in the intervention program to make it more effective. 

However, the efficiency of improved lifestyle on the prevention of progress of pre-diabetes depends on the extent of β-cell dysfunction and hyperglycemic nature, which varies from patient to patient [142].

### 4.2. Pharmacological Interventions

The progression of pre-diabetes to T2DM can also be prevented through anti-diabetic drugs or anti-obesity drugs.

#### 4.2.1. Metformin

Metformin is one of the most popular and common medications prescribed to delay the onset of diabetes. Metformin belongs to the biguanide class of anti-diabetic medication [158]. The mechanism of action of Metformin is complex and yet to be fully understood. Metformin does not get metabolized in the human body and the concentration of Metformin remains high in the liver, intestines, kidney and bladder [159,160,161]. In humans, the main site of action of Metformin is the intestines, liver and kidney [162,163]. Metformin decreases fasting plasma glucose (PG) concentration and hemoglobin A1c by suppressing liver glucose production (hepatic gluconeogenesis) or by restoring β-cell function [164,165,166,167]. This drug mainly works by shrinking the rate at which hepatic glucose is produced and enhancing the insulin action in skeletal muscles [168,169]. Two of the core mechanisms of action of this drug are via the energy sensor enzyme adenosine monophosphate-activated protein kinase (AMPK) and the inhibition of mitochondrial respiration [163]. Hepatic gluconeogenesis requires the supply of a high amount of ATP. Metformin exploits this demand of ATP to reduce hepatic gluconeogenesis. Metformin is found to inhibit the function of complex I of the mitochondrial electron transport chain. Therefore, reducing the ATP production [170,171]. One of the drawbacks, as addressed by many, regarding this mechanism of action is that a high amount of Metformin is required to observe a significant reduction in hepatic gluconeogenesis [170]. The reduction of ATP production due to the inhibition of mitochondrial respiration plays a role in the activation of AMPK as well. As the amount of ATP decreases, to maintain the energy balance, AMPK switches on pathways that produce ATP and switches off pathways that use ATP [172,173]. When Metformin is given at a smaller concentration (20 µmol/L), a longer time is needed to get significant results, in comparison to when higher concentrations are used (500 µmol/L) [174]. However, Metformin has also been found to have AMPK independent effects on hepatic gluconeogenesis. When Metformin was given to mice lacking AMPK subunits in the liver and to control mice, Metformin was found to improve glucose tolerance in both groups [175]. More of the latest studies have found that Metformin controls glucose by inhibiting fructose-1,6-bisphosphatase, the key enzyme for gluconeogenesis, rather than solely activating AMPK [163]. Another mechanism of action is that it improves the overall action of glucagon-like peptide-1 (GLP-1). Metformin increases the secretion of GLP-1 by the intestinal L-cell and/or reduces the action of the enzyme (dipeptidyl peptidase-4) that breaks down GLP-1. This improves the overall action of GLP-1 [176,177,178]. Apart from inhibiting liver gluconeogenesis, Metformin also increases glucose disposal in skeletal muscle by increasing AMPK activity in skeletal muscle of T2DM patients [179]. The increase in AMPK activity is associated with increased phosphorylation of AMPK on Thr172 and decreased acetyl-CoA carboxylase-2 activity. Hence, Metformin reduces the blood glucose level in the aforementioned ways.

In the DPP study conducted by Knowler et al. (2002), it was found that Metformin, at a dose of 2 × 850mg/day reduced the chances of development of T2DM from IGT. A total of 7.8 per 100 pre-diabetics developed diabetes when Metformin was prescribed, whereas, a total of 11.0 per 100 pre-diabetics developed the same when a placebo was prescribed. Furthermore, Metformin also improves insulin sensitivity and reduces new cases of T2DM. However, in the DPP study (Knowler et al., 2002) conducted in the USA, Metformin was found to be not as effective as lifestyle intervention [133], while in the Indian DPP conducted by Ramachandran et al. (2006) over a three years period, Metformin was found to be as effective as lifestyle intervention. The study was conducted on 531 Asian Indians, each being assigned to one of four groups (control, lifestyle modification only, Metformin only, and lifestyle modification with Metformin). The results of the lifestyle modification group and Metformin group were very similar [150]. Additionally, Knowler et al. concluded that in people with a BMI higher than 35, aged less than 65 years, and having a fasting PG higher than 110 mg/dL, Metformin reduced the chances of developing T2DM as effectively as lifestyle intervention [133]. Overall, Metformin is a relatively safe drug. Hence, it is advised to people who are aged less than 60 years, with a BMI more than 35 kg/m^2^ and with IGT or IFG, by the ADA [180]. 

#### 4.2.2. Thiazolidinediones

Thiazolidinediones target PPAR-γ. PPAR-γ are ligand activated transcription factors and activation of PPAR-γ results in insulin sensitization and increases glucose metabolism [181,182]. Thiazolidinediones work by making the adipocytes, liver and muscle cells more sensitive to insulin and by conserving the β-cell function. Insulin sensitivity is determined by the efficacy of the PPAR-γ receptors of adipocytes and muscle cells [183,184,185]. A considerable number of studies have also found that thiazolidinediones improve insulin sensitivity by decreasing plasma free fatty acid and intramyocellular lipid content. Thiazolidinediones also redistribute fats from visceral to subcutaneous adipose sites which might help to control diabetes [183,184,185]. Troglitazone, pioglitazone, and rosiglitazone are the common drugs belonging to this group and all have shown similar results in improving insulin sensitivity [186,187,188]. In the DPP, Troglitazone reduced the progression of T2DM in IGT individuals by 23% within 3 years [134]. In another study, called the Diabetes Reduction Assessment with ramipril and rosiglitazone Medication (DREAM) trial, rosiglitazone reduced the progression of T2DM in IGT individuals by 62% [135]. Pioglitazone and troglitazone have been found to control the progression of gestational diabetes as well [189,190,191]. However, rosiglitazone might have some side-effects. A double-blind study was conducted with Ramipril and rosiglitazone. Even though there was a decrease in diabetes incidence by 60%, there were a few side-effects. The side-effects included an average increase in weight by 2.2 kg, and a higher risk of the cardiovascular incident including heart failure [135,136].

#### 4.2.3. α-Glucosidase Inhibitors

This group of drugs may ameliorate T2DM by prolonging the overall carbohydrate digestion time and decreasing the rate of glucose absorption [192]. In the STOP Non-Insulin-Dependent Diabetes Mellites (STOP-NIDDM) trial, over a 3.3 year follow up, acarbose reduced the progression of IGT to T2DM by 25% [193,194]. In another study in Japan, voglibose reduced diabetes progression by 40% in a period of 48 weeks. However, both studies have shown notable side effects among the individuals. The major side effects were related to the gastrointestinal system that included flatulence and diarrhea. In the STOP-NIDDM study and the Japanese study, 37% and 7% participants respectively terminated the trial before the completion of the program due to the side effects [193,194,195]. Although α-glucosidase inhibitors alone can improve glycemic variability, it should be noted that they do not improve insulin sensitivity [196]. Combination with other groups of anti-diabetic drugs may be necessary to effectively inhibit the progression of T2DM.

#### 4.2.4. Incretins

Gastric inhibitory polypeptide (GIP) and glucagon-like peptide 1 (GLP-1) are two of the major stimuli for insulin secretion. GLP-1 slows down diabetes progression by reducing glucagon secretion, keeping the stomach full for a longer period, and shrinking the appetite. This limits food intake and leads to weight loss. However, both GIP and GLP-1 get broken down by Dipeptidyl Peptidase-IV (DPP4). Therefore, GIP and GLP-1 cannot be used as a means of therapy for preventing diabetes progression. To overcome this hurdle, a GLP-1 receptor agonist is used. Two such common agonists are liraglutide and exenatide. These agonists are not broken down by DPP4 and can accomplish similar results to GLP-1. In a study, exenatide was found to be improving β-cell function and playing a role in reducing weight [197]. However, no study has yet been carried out to track the effect of GLP-1 on the progression of IGT to T2DM. But a study over a period of 2 years has been conducted on obese individuals, and exenatide and liraglutide have been shown to cause weight loss [198].

#### 4.2.5. Sodium-Glucose Cotransporter (SGLT) 2 Inhibitors

SGLT 2 inhibitors are one of the latest additions to pharmacological interventions in treating pre-diabetes and T2DM. Canagliflozin, dapagliflozin and empagliflozin are the common approved drugs belonging to the class of SGLT 2 inhibitors [44,199,200]. SGLT 2 inhibitors can be recommended alone or in combination with other glucose controlling medications [201]. The doses of different SGLT 2 inhibitors are different [202]. Some of the most common side effects of this class of medication is urogenital tract infection, genital infection, breast and bladder cancer [203,204]. Because of all these, more serious research needs to be carried out to verify the safety of this class of medication. SGLT 2 inhibitors mainly work by interfering with the SGLT 2 in the kidneys. In a healthy individual, glucose is filtered and reabsorbed by the kidneys. SGLT 2 is responsible for the reabsorption of glucose in the kidney [205]. In pre-diabetics and diabetes, the expression of SGLT 2 increases, resulting in more reabsorption of glucose by the kidney [206]. When SGLT 2 inhibitors are used, they tend to decrease this reabsorption of glucose and reduce FPG and HbA1c [207]. Therefore, this increases urinary glucose elimination and decreases blood glucose level. 

#### 4.2.6. Anti-Obesity Drugs

Orlistat is an anti-obesity drug which works by reducing the absorption of fat by almost 30%. In a study over a period of 1.5 years, it was found that the progression of IGT to diabetes was reduced when orlistat was given to individuals while the subjects maintained a low-calorie diet and lost weight. According to the XENial in the Prevention of Diabetes in Obese Subjects (XENDOS) trial, orlistat reduced diabetes development by 37% over a 4-year treatment [208].

### 4.3. Bariatric Surgery

Bariatric surgery mainly works by targeting the calorie intake of the subject which ultimately results in weight loss. As reviewed by Bansal, the most common modalities under bariatric surgery include Roux-en-Y gastric bypass, laparoscopic adjustable gastric banding, sleeve gastrectomy, and duodenal switch with biliopancreatic diversion [209]. 

A Swedish Obese Study conducted by Sjostrom et al., tracked the weight of subjects who underwent a bariatric surgery and compared the data with the control group. A total of 4047 individuals were followed up after two years, and 1703 individuals were followed up after ten years. There was a 23.4% and 16.1% decrease in weight for the 2 years and 10 years follow up groups respectively. On the other hand, there was an increment in weight for the control group. There was a 0.1% increase after 2 years follow up, and a 1.6% increase after 10 years follow up [210]. Overall, there was a 75% reduction in risk for developing diabetes after bariatric surgery was performed [209,210]. In another study reported by Pories et al., it was found that 78% of diabetic and 98% of pre-diabetic subjects restored normal glucose level after gastric bypass surgery [211]. Therefore, bariatric surgeries provide promising methods for the treatment of pre-diabetes as they target obesity, which is one of the major risk factors of diabetes.

## 5. Complications

**Figure 1:**
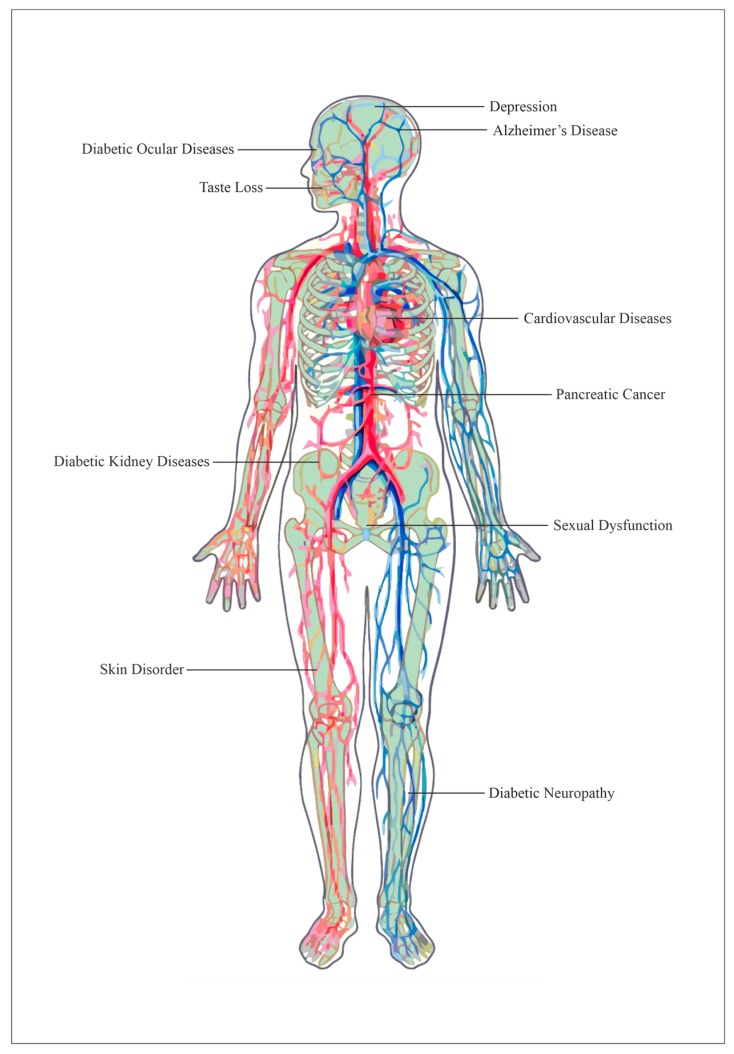
A cross-section image of the parts of human body that can be affected by diabetic complications. Image of human body generated using Chembiodraw (v. Ultra 14.0).

Pre-diabetes progresses to diabetes if left untreated. Moreover, if diabetes is left unmanaged and untreated, various chronic complications can develop, increasing the diabetic patient’s discomfort and could even lead to death. A list of some known diabetes complications that have been previously researched or reported on is summarized in Figure 1.

### 5.1. Cancer

Cancer is a genetic disease resulted from both internal factors and external factors [212,213]. It is a less-explored complication that can result from diabetes. With 80% of pancreatic cancer patients presenting with impaired glucose tolerance, and because one major pathophysiology of diabetes is the impaired β-cell function, there appears to be an association between T2DM and pancreatic cancer [214]. While there are many studies that establish diabetes as a risk factor of pancreatic cancer, where 1% to 2% of patients who had recently suffered from diabetes were reported to develop pancreatic cancer in 3 years [215], this relationship also works vice versa, such that pancreatic cancer can be the cause of diabetes. In fact, type 3 diabetes is a form of diabetes that is caused by the loss of function of the pancreas, which occurs in up to 30% of pancreatic cancer patients [215]. In addition to pancreatic cancer, diabetes can increase the risks, by twice or more, of developing liver cancer—since the liver is also exposed to high levels of insulin for diabetic patients—and endometrium cancer, as well as increase the risk of developing cancers of colon and rectum, breast, and bladder by 1.2 to 1.5 times [216]. However, in two meta-analysis on diabetes and prostate cancer, diabetes lowered the risk of developing prostate cancer by 9% [217] and 16% [218]. This is further affirmed by a case study conducted by Coker et al. which saw a stronger protective effect against prostate cancer in patients suffering from diabetic complications and also for African American men, although the study concurred that more research is still required to establish the biological mechanisms between diabetes and prostate cancer risk [219].

### 5.2. Depression

Depression is a psychological complication that can result from diabetes, and the relationship between the former and the latter is often regarded to be bi-directional. While different studies report different levels of risk of diabetic patients developing depression, it is generally more likely for diabetic individuals to be found depressed, as compared to non-diabetic individuals under the same settings [220,221]. Complex mechanisms behind the association of diabetes and depression remain understudied. However, it has been suggested that the consumption of atypical antipsychotic medications [221], the patient’s sufferings caused by advanced diabetes, as well as diabetic abnormalities in neurohormonal and neurotransmitter function [220] could be reasons for this association. Conversely, depression is a risk factor that can rivals other factors such as smoking [221] in causing diabetes, as depression makes it difficult for diabetic patients to adhere to medical treatments and glycemic controls [220], thereby furthering diabetes complications.

### 5.3. Alzheimer’s Disease

Recent studies proposed Alzheimer’s disease to be “Type 3 Diabetes” resulting from impaired insulin signaling [222]. Arvanitakis et al. have concluded in their study that there is a 65% higher risk level for diabetic patients to develop Alzheimer’s disease than non-diabetic individuals [223]. The relationship between the two, however, is uncertain. Moreover, in a review on Alzheimer’s disease and diabetes, it was suggested that Type 2 diabetes itself was not sufficient to cause Alzheimer’s disease, although diabetes could have served as a pathogenesis and progression co-factor [222].

### 5.4. Diabetic Ocular Diseases

There are several types of ocular diseases that can arise from diabetes, eventually causing vision loss. Among the numerous ocular complications, most research focuses on diabetic retinopathy, since even the World Health Organization estimated it to cause blindness in 5% of blind people [224]. Moreover, more than 60% of Type 2 diabetes patients develop some form of diabetic retinopathy within a decade of diabetes incidence [225] and nearly all Type 1 diabetes patients suffer this two decades after puberty [226]. Although the pathophysiological cause behind diabetic retinopathy is unclear, several mechanisms have been proposed. Nine biochemical pathways that are the main contributors to diabetic retinopathy have been extensively reviewed by Tarr et al. [224]. The pathways that associate retinopathy with diabetes, reviewed by Tarr et al. include: an increased polyol pathway, activation of protein kinase C, increased expression of growth factors, hemodynamic changes, accelerated formation of advanced glycation end-products, oxidative stress, activation of the renin–angiotensin–aldosterone system, subclinical inflammation and capillary occlusion [224]. Other less commonly known diabetic ocular diseases that are known to directly cause, link and possibly link to diabetes have also been previously classified by Jeganathan et al. [227] and are summarized as follows (Table 3):

### 5.5. Taste Loss

Rarely are diabetes complications associated with taste. However, altered taste and a preference for sweet food were presented in a case study by Bhandare et al. where a newly diagnosed diabetes patient was found to suffer from blunted taste, which could partially be reversed when hyperglycemia was corrected. Therefore, altered taste has been proposed as an indicator of blood sugar fluctuations [228].

### 5.6. Cardiovascular Diseases

Cardiovascular diseases are the main contributor of death and disability in diabetic patients [229,230]. Cardiovascular diseases were reported to account for more than 50% of deaths, by Einarson et al., after they reviewed the prevalence of cardiovascular diseases in Type 2 diabetic patients, recorded in 57 studies published from 2007 to 2017 [230]. More specifically, the American Heart Association regarded diabetes to be a risk, rather than a risk factor, of coronary heart diseases after recent studies showed that the risk of having myocardial infarction in diabetic individuals equals that of patients who had a history of previous myocardial infarction [70]. Additionally, while Type 2 diabetic patients showed an increased risk of 15–400% of developing stroke [70], Type 1 diabetic patients suffer higher mortality from ischemic heart disease, as compared to the rest of the population [70]. This is because numerous cardiovascular diseases risk factors are present in diabetic patients such as obesity, hypertension, and dyslipidemia [229,231]. Moreover, diabetes can also contribute to cardiomyopathy as well as coronary artery disease directly, rather than indirectly through the risk factors [231].

### 5.7. Diabetic Kidney Diseases

Diabetic kidney disease or diabetic nephropathy is another common microvascular diabetic complication where it has been estimated to be prevalent in 30% and 40% of Type 1 and Type 2 diabetic patients respectively [232]. Diabetic nephropathy was characterized by Nazar C.M.J. as having protein albumin found in the urine, coupled with a decrease in glomerular filtration rate and an increase in arterial blood pressure [233]. The development of diabetic nephropathy, similar for both types of diabetes, starts with albuminuria or known as incipient nephropathy—moving from microalbuminuria to macroalbuminuria [234]—then to overt nephropathy, eventually leading to end-stage renal disease [235]. In fact, diabetes is known to become the most frequent cause of end-stage renal disease globally, where countries such as Malaysia, Mexico, and Singapore see diabetes being the main cause of end-stage renal disease in 60% of their diabetic patients [236]. While end-stage renal disease develops in 50% and more than 75% of Type 1 diabetic patients after they develop overt nephropathy 10 and 20 years later respectively, only 20% progress from overt nephropathy after 20 years for Type 2 diabetic patients [235]. Moreover, it should be noted that the incidence of diabetic nephropathy is higher and developed at a faster rate in Type 2 than Type 1 diabetic patients, as Type 2 diabetic patients often have their diagnosis delayed [235].

### 5.8. Sexual Dysfunction

Higher prevalence of sexual dysfunction as a complication arising from diabetes have been reported in both men and women, although the association is less conclusive for women [237]. While sexual dysfunction for men results from more physiological reasons, female sexual dysfunction was found to be more related to social and psychological reasons [237]. Among the various sexual problems, erectile dysfunction was the most common dysfunction in men [238], with diabetic men experiencing it three times more than non-diabetic men [239], where the association between erectile dysfunction and diabetes is multifactorial [240]. For women, a few types of sexual dysfunction have been reported, such as lower sexual desire, genital arousal disorder, orgasmic disorder and pain [239]. Additionally, although a study presented by Ahmed et al. concluded that female sexual dysfunction was more prevalent in Type 1 than Type 2 diabetic women in Egypt [241], there is still generally a lack of studies in comparing the prevalence of sexual dysfunction between the two types of diabetes [237,238].

### 5.9. Skin Disorder

A complication of diabetes that is highly prevalent, but often goes undiagnosed is skin disorders, with its reported prevalence at 51–97% in both Type 1 and Type 2 diabetes in different regions worldwide [242]. Although various studies presented different statistical data on skin disorder in diabetic patients, cutaneous infection, xerosis, and inflammatory skin diseases were the most common types of skin disorders reported for diabetic individuals [242,243]. Skin disorders usually occur in poorly controlled diabetic patients, which is expected, since diabetic skin disorders are found to be highly correlated to glycemic control [242].

### 5.10. Diabetic Neuropathy

Diabetic neuropathy is the most common complication of diabetes, for both Type 1 and Type 2 since it has been reported in 90% of diabetic patients [244]. Among the various neuropathy syndromes, the most common syndrome is diabetic polyneuropathy, which accounts for 75% of the syndromes [245]. Diabetic polyneuropathy is defined as a peripheral nerve dysfunction [246] and is the main contributor to disability in diabetes, stemming from foot ulceration and amputation, gait disturbance, and fall-related injury [245]. Pain, another main symptom of diabetic neuropathy [244] is experienced by 20–30% of patients suffering from diabetic polyneuropathy [246]. Diabetic neuropathic pain is characterized by Schreiber et al. as a “tingling, burning, sharp, shooting, and lancinating or even as electric shock sensations” which often worsen in the night [244]. Pain can also be a complication that is associated with depression, as mentioned earlier, as it decreases the quality of life and influences the mood of diabetic patients [244]. Moreover, the pathological reasoning behind diabetes and pain has yet to be fully established [244,245].

## 6. Conclusions

With every nation’s attention on diabetes mellitus, there is an urgent need for a multidimensional insight into this disease. In this review, many possible genetic and epigenetic markers as well as lifestyle and environmental factors have been explored to provide a better understanding of the risk factors of T2DM. Simultaneously, we have discussed the novel diagnostic methods of T2DM for a more sensitive and specific approach towards identifying diabetic patients and pre-diabetic population. Different treatment options have been comprehended and included in this review for comparison among the treatment regimes. Additionally, some of the more common complications resulting from diabetes have also been reviewed. By establishing this thorough review on diabetes, we hope to provide invaluable insight as to how we can combat diabetes and pre-diabetes around the world.

## Figures and Tables

**Table 1:** A comparison between normal, pre-diabetes and diabetes based on three diagnosis methods.

	FPG	PG in OGTT	A1C
**Normal**	<100 mg/dL or 5.5 mmol/L	<140 mg/dL or 7.8 mmol/L	<5.7% or 39 mmol/mol
**Pre-Diabetes**	≥100 mg/dL or 5.5 mmol/L	≥140 mg/dL or 7.8 mmol/L	≥5.7% or 39 mmol/mol
**Diabetes**	≥126 mg/dL or 7.0 mmol/L	≥200 mg/dL or 11.1 mmol/L	≥6.5% or 48 mmol/mol

**Table 2:** Summary of some genes responsible for diabetes mellitus.

Year of Genome Wide Significance (GWS)	Locus	Marker	Chr	Type of Mutation	Encoded Protein of Wild Type	Trait
2000	*PPARG*	rs1801282 [96]	3	Missense: Pro12Ala	PPAR-γ ^a^	T2DM
2003	*KCNJ11*	rs5219 [97]	11	Missense: Glu23Lys	Kir6.2 of pancreatic β-cells ^a^	T2DM
2007	*CDKAL1*	rs7754840 [107,108]	6	Intronic	CDK5 regulatory subunit-associated protein 1-like 1 [109]	T2DM
	*SLC30A8*	rs13266634 [110,111]	8	Missense: Arg325Trp	Islet-specific zinc membrane transporter (ZnT8) ^a^	T2DM and FG
	*IGF2BP2*	rs4402960 [107,108]	3	Intronic	Insulin-like growth factor 2 mRNA-binding protein ^a^	T2DM
	*CDKN2A/B*	rs10811661 [107,108]	9	25 kb upstream	p16 (INK4A) ^a^	T2DM

FG = Fasting Glucose. ^a^ All of the information has been retrieved from “Genetics Home Reference” (https://ghr.nlm.nih.gov). Part of this table is adapted from [112].

**Table 3:** Table showing ocular diseases other than diabetic retinopathy that can arise as complications of diabetes, adapted from the review by Jeganathan et al. [227].

Direct Causes	Known Links	Possible Links
Development of Cataracts	Glaucoma	Retinal vein occlusion
Ischemia caused by anterior optic nerve	Ocular ischemic syndrome	Retinal arteriolar emboli
Diabetic papillopathy		Retinal artery occlusion
Ocular movement disorders		Corneal diseases
